# Observed and predicted ages at peak height velocity in soccer players

**DOI:** 10.1371/journal.pone.0254659

**Published:** 2021-07-26

**Authors:** Robert M. Malina, Manuel J. Coelho-e-Silva, Diogo V. Martinho, Paulo Sousa-e-Siva, Antonio J. Figueiredo, Sean P. Cumming, Miroslav Králík, Sławomir M. Kozieł

**Affiliations:** 1 Department of Kinesiology and Health Education, University of Texas, Austin, Texas, United States of America; 2 University of Louisville, School of Public Health and Information Sciences, Louisville, Kentucky, United States of America; 3 University of Coimbra, FCDEF, Coimbra, Portugal; 4 University of Coimbra, CIDAF (uid/dtp/04213/2020), Coimbra, Portugal; 5 Department of Health, University of Bath, Bath, United Kingdom; 6 Faculty of Science, Department of Anthropology, Masaryk University, Brno, Czech Republic; 7 Department of Anthropology, Polish Academy of Sciences, Hirszfeld Institute of Immunology and Experimental Therapy, Wrocław, Poland; Universidade Federal de Mato Grosso do Sul, BRAZIL

## Abstract

The purpose of the study was to evaluate predicted maturity offset (time before age at PHV) and age at PHV (chronological age [CA] minus maturity offset) in a longitudinal sample of 58 under-13 club level soccer players in central Portugal for whom ages at PHV were estimated with the SITAR model. Two maturity offset prediction equations were applied: the original equation which requires CA sitting height, estimated leg length, height and weight, and a modified equation which requires CA and height. Predicted maturity offset increased, on average, with CA at prediction throughout the age range considered, while variation in predicted maturity offset and ages at PHV within CA groups was considerably reduced compared to variation in observed ages at offset and at PHV. Predicted maturity offset and ages at PHV were consistently later than observed maturity offset and age at PHV among early maturing players, and earlier than observed in late maturing players. Both predicted offset and ages at PHV with the two equations were, on average, later than observed among players maturing on time. Intra-individual variation in predicted ages at PHV with each equation was considerable. The results for soccer players were consistent with similar studies in the general population and two recent longitudinal studies of soccer players. The results question the utility of predicted maturity offset and age at PHV as valid indicators of maturity timing and status.

## Introduction

Predicted maturity offset, defined as time before peak height velocity (PHV) [1,2], and estimated age at PHV, i.e., chronological age (CA) minus predicted offset, are widely used as estimates of maturity status (state of maturation at the time of observation) and/or timing (age at which a specific maturational event occurs) in studies of youth athletes and to a lesser extent in studies youth physical activity and fitness [3–6]. The original sex-specific equations require CA, sitting height, estimated leg length, height and weight [1], while the modified equations [2] require CA and height (both sexes) or sitting height (boys). Validation studies of the original equations in three independent longitudinal series, the Wroclaw Growth Study [7,8], the Fels Longitudinal Study [9] and the Cracow Growth Study [10], and of the modified equations in two of the samples [10,11] have indicated major limitations of the predictions in both males and females. The validity of the prediction equations has also been questioned in longitudinal samples of female artistic gymnasts [12] and soccer players [13,14], but sample sizes in longitudinal samples tend to be limited and to some extent select as they are limited to athletes who have persisted in the respective sports [15]. Similarly, cross-sectional studies of tennis [16] and soccer [17] players have questioned maturity status classifications based on the original prediction equations [1] relative to classifications based on skeletal age.

Current interest in the application of the maturity offset prediction equations in samples of youth athletes is considerable [3–6]. Predictions based on the original and modified equations, however, depend upon CA and body size at prediction, have reduced variation relative to observed ages at PHV, and have major limitations with early and late maturing youth as defined by observed ages at PHV [7–11]. The latter are problematic as advanced (early for CA) or delayed (late for CA) maturity status is often of major concern in developmental studies of youth athletes [15].

In the context of the preceding, the purpose of the present study is to evaluate predicted maturity offset and derived ages at PHV with the original [1] and modified [2] equations in a sample of 58 soccer players for whom age at PHV was determined from longitudinal height records. The study specifically considers variation in the predictions in three contexts: (i) relative to actual maturity offset and observed age at PHV at each observation, (ii) among players differing in the timing of observed age at PHV, and (iii) within individual players.

## Materials and methods

### Research design and procedures

The data set for the present study was extracted from the *Coimbra Soccer Longitudinal Project* [18]. This project was conducted according to the standards established by the declaration of Helsinki [19], and formal approval was obtained from the *University of Coimbra Sports Sciences and Physical Education Board* (FCDEFUC/AAC/2003; FCDEFUC/ADCA/2003; FCDEFUC/CFM/2003; FCDEFUC/CFUC/2003; FCDEFUC/GRVM/2003). Signed institutional agreements were also obtained from the Presidents of the respective clubs. All players were registered with the *Portuguese Soccer Federation*. Male players 11–14 years of age were recruited from five clubs in the Midlands of Portugal; the initial sample included 159 players [20]. Written consent was obtained from parents or legal guardians of the participants who were informed that contribution to the study was voluntary and that they could withdraw from the study at any time. All observations were completed at the *Biokinetics Laboratory* of the *Coimbra University Stadium*.

### Sample

According to the *Portuguese Soccer Federation*, male soccer players were grouped as infantiles (aged 11–12 years, n = 87) and initiates (aged 13–14 years, n = 72). The analysis in the current study is limited to under-13 players (U13) who were measured annually in December for four or five years (n = 59). CAsat baseline ranged from 10.98 to 12.94 years. The heights of one player were not successfully modeled; consequently, the final sample was composed of 58 players. All players were of European ancestry, except one. Participants trained and competed September through May. They had a median of 3 years of soccer experience at baseline (range: 2–6 years). The clubs had 3–5 training sessions per week (each about 90–120 minutes) and usually one game, mainly on Saturdays.

### Anthropometry

Participants wore shorts and a t-shirt; shoes were removed. Height and sitting height were measured to the nearest 0.1cm using, respectively, a stadiometer (Harpenden 98.603, Holtain Ltd, Croswell, UK) and a table (Harpenden sitting height table, model 98.607, Holtain Ltd, Crosswell, UK). Body weight was measured to the nearest 0.1 kg using a scale (SECA 770, Hanover, MD, USA). The heights, sitting heights and weights of players who continued at the respective clubs were subsequently measured on an annual basis. Measurements were made by a single observer (MJCS). Intra-observer technical errors of measurement for height, sitting height and weight were 0.27 cm, 0.31 cm and 0.47 kg, respectively [19].

### Age at PHV

The longitudinal height records were fit with the Superimposition by Translation and Rotation (SITAR) model [21–24] to derive an age at peak height velocity for each player. As noted, the heights of one player were not successfully modeled; the estimated age at PHV for the player was outside of the empirical data range and was inconsistent with his advanced skeletal maturity status at observations one and three. Mean age at PHV for the remaining 58 players was 13.60±0.85 years, with a range from 11.89 to 15.49 years [25].

### Predicted maturity offset

Maturity offset, defined as time before PHV, was predicted at each observation for the 58 players with the original equation for boys [1]:

Maturityoffset(years)=−9.236+(0.0002708×(LegLength×SittingHeight))+(−0.001663×(CA×LegLength))+(0.007216×(CA×SittingHeight))+(0.02292×(WeightbyHeightRatio×100)) Eq (1)

Leg length was estimated at each observation as standing height minus sitting height. The need to multiply the weight by height ratio by 100 was overlooked in the original report [1]; in some publications using the equation, it is not clear if the adjustment was applied. Maturity offset was also predicted at each observation with a modified equation for boys that incorporated age and height [2]:

Maturityoffset(years)=−7.999994+(0.0036124×(CA×Height)) Eq (2)

The equation with age and height was selected for evaluation as it is increasingly used [26–31]. Standard errors for the original [1] and modified [2] equations were, respectively, 0.592 and 0.542 year. Predicted maturity offset and predicted age at PHV with the respective equations are subsequently labelled in the text, tables and figures as Mirwald and Moore, respectively.

### Predicted age at PHV

Predicted age at PHV was estimated as CA minus predicted maturity offset at each observation for individual players with the respective equations.

### Observed maturity offset

Observed or actual maturity offset at each observation was estimated as CA at prediction minus observed age at PHV based on the SITAR model.

### Analyses

Descriptive statistics were calculated at each observation for CA and actual offset, for predicted maturity offset and age at PHV, and for the difference of predicted age at PHV minus observed age at PHV with the two prediction equations. The players were also classified as advanced (early), average (on time) or delayed (late) maturing relative to the mean and standard deviation for age at PHV (SITAR) in the total sample. Average was defined as an age at PHV within ±1 SD of the mean age at PHV for the total sample of 58 players (13.60±0.85 years); delayed was a PHV >14.45 years and advanced was a PHV <12.75 years.

Linear mixed-effect models with the data grouped by subjects (random effects) were used with a maximum-likelihood estimator to evaluate the variance structure of the dependent variable, i.e., the differences between observed and predicted ages at PHV. Separate analyses were done for predictions with the Mirwald and Moore equations. The difference of the dependent variable from zero (so-called *unconditional means* model) was initially tested. In the second step, the effect of predictions at observations 1–5 (as a time variable) on the dependent variable at the population level (fixed effect) and at the intra-individual level (i.e., random slopes model, or *unconditional growth* model) were tested. Note that the term growth in the statistical context refers to the general change in a dependent variable with a time variable; it does not refer to growth in the biological sense. Finally, maturity status was added as a fixed factor to test the effect of variation in maturity timing on the predictions. The model was run separately for predictions with the Mirwald and Moore equations in the R-software [22] with the help of the *nlme* statistical package [32].

Weighted Deming regression using the jackknife procedure [33] was used to compare observed age at PHV estimated with the SITAR model (i.e., the reference) with predicted ages at PHV at each observation based on the two equations. The weighted Deming procedure considers both *x* (observed age at PHV) and *y* (predicted age at PHV) as subject to measurement error whereas simple regression permits only the *y* variable to have an associated error. With the weighted Deming regression, systematic differences between *x* and *y* are indicated by the intercept, while proportional differences are indicated by the slope.

Many applications of the equations use predicted maturity offset to classify youth as pre-PHV, at/circa/mid-PHV, or post-PHV using a band of -0.5 to +0.5 year to define the interval at PHV [29,34–41]; a band of -1.0 to +1.0 year is used less often [42–46]. On the other hand, some studies do not report the specific cut-offs that were used [47–49]. The standard errors of the prediction equations, 0.592 and 0.542 year, also approximate the narrow cut-offs used in many studies. Thus, the number and percentage of predicted ages at PHV with each equation within ±0.5 year of observed age at PHV at each observation were estimated for players of contrasting maturing status and for the total sample.

## Results

Descriptive statistics for CA at prediction, observed and predicted maturity offset, predicted ages at PHV and the difference of predicted age at PHV minus observed age at PHV (the reference for comparison) with the original (Mirwald) and modified (Moore) equations in the soccer players are summarized in Table 1. Observed and predicted maturity offset increase linearly, on average, across the five observations. Predicted offset with the Moore equation is similar to actual offset at observation one but is then less than actual offset at subsequent observations. Predicted maturity offset with the Mirwald equation is less than actual offset and predicted offset with the Moore equation across the five observations.

**Table 1:** Sample sizes and descriptive statistics for chronological age (CA) at prediction, observed maturity offset and predicted maturity offset and ages at PHV, and the difference of predicted age at PHV minus observed ages at PHV (criterion) with the original (Mirwald) and modified (Moore) equations in soccer players at each observation†.

Observations	n	CA (years)	Maturity Offset (yrs)			Predicted age at PHV (yrs)		Predicted minus Observed age at PHV (yrs)	
			Observed	Predicted					
				Mirwald	Moore	Mirwald	Moore	Mirwald	Moore
1	58	11.9±0.5	-1.72±0.86	-2.09±0.51	-1.78±0.49	13.97±0.36	13.66±0.27	0.37±0.71	0.06±0.74
2	58	12.9±0.5	-0.70±0.86	-1.26±0.61	-0.93±0.56	14.15±0.45	13.82±0.35	0.56±0.63	0.23±0.68
3	58	13.9±0.5	0.29±0.86	-0.26±0.71	-0.01±0.57	14.14±0.51	13.90±0.37	0.55±0.56	0.30±0.64
4	55	14.9±0.5	1.33±0.86	0.71±0.66	0.89±0.55	14.17±0.52	13.99±0.36	0.62±0.59	0.44±0.72
5	40	15.9±0.5	2.34±0.87	1.63±0.52	1.72±0.50	14.25±0.43	14.16±0.32	0.71±0.82	0.65±0.92

†Observed (actual) maturity offset was calculated as CA at prediction minus observed age at PHV for each player, see text for details.

Corresponding trends in predicted ages at PHV and the difference of predicted minus observed ages at PHV parallel those for maturity offset. Standard deviations for predicted maturity offset and ages at PHV with both equations are consistently lower than corresponding standard deviations for observed offset and age at PHV across the five observations. Variability is reduced more so with the Moore compared to the Mirwald equation.

Results of the mixed effects model indicate significant differences between observed and predicted ages at PHV with the Mirwald equation (F = 40.95; p<0.001) and also with the Moore equation (F = 9.39; p<0.01). Details of the analytical protocol and results are summarized in S1 Table. The differences between observed and predicted ages at PHV with the respective equations at each observation increase significantly with subsequent observations with the Mirwald (F = 22.81; p<0.001) and the Moore (F = 172.97; p<0.001) equations, although the 95% confidence intervals indicate that the difference at observation 1 for the Moore equation is not different from zero.

Intercepts based on weighted Deming regressions for the two prediction equations are well above zero at each observation, indicating that the methods differ significantly by a constant error (Table 2). Estimated slopes for each regression are <1.0, indicating significant proportional differences between predicted ages at PHV with each equation and observed age at PHV. Overall, the results indicate systematic error for predicted ages at PHV.

**Table 2:** Intercepts and slopes, and respective standard errors (SE) and 95% confidence limits based on the weighted Deming regression of predicted ages at PHV (y-axis) and observed (actual) ages at PHV (x-axis) for the Mirwald and Moore prediction equations at each observation (Obs) in youth soccer players*.

Obs	Mirwald						Moore					
	Intercept			Slope			Intercept			Slope		
	value	SE	(95% CL)	value	SE	95% CL)	value	SE	(95% CL)	value	SE	(95% CL)
1	10.26	0.77	(8.75; 11.81)	0.27	0.06	(0.15; 0.39)	11.17	0.53	(10.10; 12.24)	0.18	0.04	(0.10; 0.26)
2	8.35	0.94	(6.46; 10.24)	0.43	0.07	(0.28; 0.57)	9.86	0.67	(8.52; 11.20)	0.29	0.05	(0.19; 0.39)
3	7.01	0.77	(5.45; 8.57)	0.52	0.06	(0.41; 0.64)	9.36	0.62	(8.11; 1.62)	0.33	0.05	(0.24; 0.43)
4	7.26	0.77	(5.72; 8.79)	0.51	0.06	(0.40; 0.62)	10.49	0.76	(8.98; 12.01)	0.26	0.06	(0.15; 0.37)
5	11.18	1.07	(9.01; 13.35)	0.23	0.08	(0.06; 0.39)	13.58	0.97	(11.62; 15.54)	0.04	0.07	(-0.10; 0.19)

*All intercepts and slopes are significant in showing, respectively, systematic and proportional differences between predicted and observed ages at PHV with each equation.

Descriptive statistics for the three maturity groups are summarized in Table 3. Sample sizes and ages at PHV of players in each maturity timing group were as follows: advanced, n = 8, 12.30±0.27 years; average, n = 38, 13.50±0.51 years; delayed, n = 12, 14.76±0.27 years.

**Table 3:** Sample sizes and descriptive statistics (mean ± standard deviation) for chronological age (CA) at prediction, observed maturity offset and predicted maturity offset, predicted ages at PHV and the difference of predicted age at PHV minus observed ages at PHV (criterion) with the original (Mirwald) and modified (Moore) equations at each observation in players classified as advanced, average and delayed based on observed ages at PHV^†^.

Obs	n	CA (yrs)	Maturity Offset (years)			Predicted APHV (years)		Predicted minus Observed APHV (years)	
			Observed	Predicted	Mirwald	Moore	Mirwald	Moore
				Mirwald					Moore
**Early**									
1	8	11.4±0.5	-0.90±0.44	-2.14±0.63	-1.94±0.64	13.54±0.29	13.34±0.22	1.24±0.26	1.04±0.34
2	8	12.4±0.5	0.02±0.44	-1.14±0.70	-0.98±0.72	13.55±0.36	13.40±0.33	1.25±0.35	1.10±0.42
3	8	13.4±0.5	1.11±0.44	-0.13±0.68	-0.04±0.68	13.54±0.37	13.45±0.31	1.24±0.32	1.15±0.40
4	8	14.4±0.5	2.11±0.44	0.81±0.55	0.76±0.65	13.59±0.26	13.64±0.32	1.29±0.23	1.34±0.43
5	7	15.4±0.5	3.10±0.47	1.45±0.55	1.35±0.64	13.92±0.25	14.02±0.29	1.65±0.17	1.75±0.38
**Average**									
1	38	11.9±0.5	-1.57±0.76	-2.05±0.54	-1.73±0.49	13.98±0.32	13.66±0.24	0.48±0.53	0.16±0.53
2	38	12.9±0.5	-0.56±0.76	-1.21±0.64	-0.87±0.57	14.16±0.37	13.82±0.27	0.65±0.50	0.32±0.48
3	38	13.9±0.5	0.43±0.76	-0.15±0.72	0.08±0.57	14.08±0.39	13.86±0.27	0.58±0.46	0.36±0.48
4	37	14.9±0.5	1.46±0.75	0.85±0.63	1.00±0.50	14.09±0.35	13.94±0.28	0.61±0.55	0.46±0.58
5	26	15.9±0.5	2.39±0.87	1.70±0.49	1.77±0.40	14.23±0.37	14.16±0.31	0.69±0.72	0.62±0.72
**Late**									
1	12	12.0±0.4	-2.73±0.26	-2.18±0.38	-1.83±0.38	14.21±0.29	13.85±0.22	-0.55±0.34	-0.90±0.30
2	12	13.0±0.4	-1.72±0.26	-1.51±0.39	-1.09±0.39	14.55±0.30	14.13±0.23	-0.21±0.36	-0.63±0.31
3	12	14.0±0.4	-0.73±0.26	-0.70±0.50	-0.28±0.46	14.73±0.32	14.31±0.24	-0.03±0.38	-0.45±0.34
4	10	15.0±0.4	0.22±0.22	0.08±0.53	0.57±0.54	14.91±0.35	14.42±0.28	0.14±0.44	-0.35±0.43
5	7	16.2±0.4	1.40±0.26	1.58±0.62	1.88±0.57	14.63±0.51	14.33±0.37	-0.17±0.49	-0.48±0.47

Obs (observations)

†Players were classified as late, average or early maturing on the basis of their observed age at PHV (SITAR model) relative to age at PHV for the total sample of soccer players- see text for details. Observed (actual) maturity offset was calculated as CA at prediction minus observed age at PHV for each player.

Results of the mixed-effects model comparing the three maturity groups indicate that maturity status as a fixed factor has a significant effect on the dependent variable (predicted ages at PHV) with both the Mirwald (F = 36.85, p<0.001) and the Moore (F = 51.28, p<0.001) equations. By inference, predicted ages at PHV differ relative to observed age at PHV in each group. However, the interaction between observation and maturity group is significant for the Mirwald equation (F = 5.39, p = 0.005) and indicates different slopes of change in predicted ages at PHV with consecutive observations in the maturity groups. The latter reflects the trend in differences between predicted and observed ages at PHV for the Mirwald equation which are not significant except at observation 1. In contrast, the interaction term is not significant for the Moore equation.

In the context of the results of the mixed-effects model, differences between predicted and observed ages at PHV are significant and positive across the five observations among players advanced in maturity timing (i.e., early ages at PHV). The predicted ages at PHV are consistently later observed ages at PHV.

Results are similar for players maturing on time (average), i.e., predicted ages at PHV with the two equations are later than observed age at PHV. Across the five observations, the differences between predicted and observed ages at PHV with each equation are significant, although predicted age at PHV with the Moore equation at observation one approaches that for observed age at PHV.

Among late maturing players, in contrast, differences between predicted and observed ages at PHV for the Mirwald equation are not significant except at observation one, while differences between predicted and observed ages at PHV for the Moore equation are significant at each observation. The differences between predicted and observed ages at PHV with the Mirwald equation are also smaller than corresponding differences with the Moore equation.

**Fig 1:**
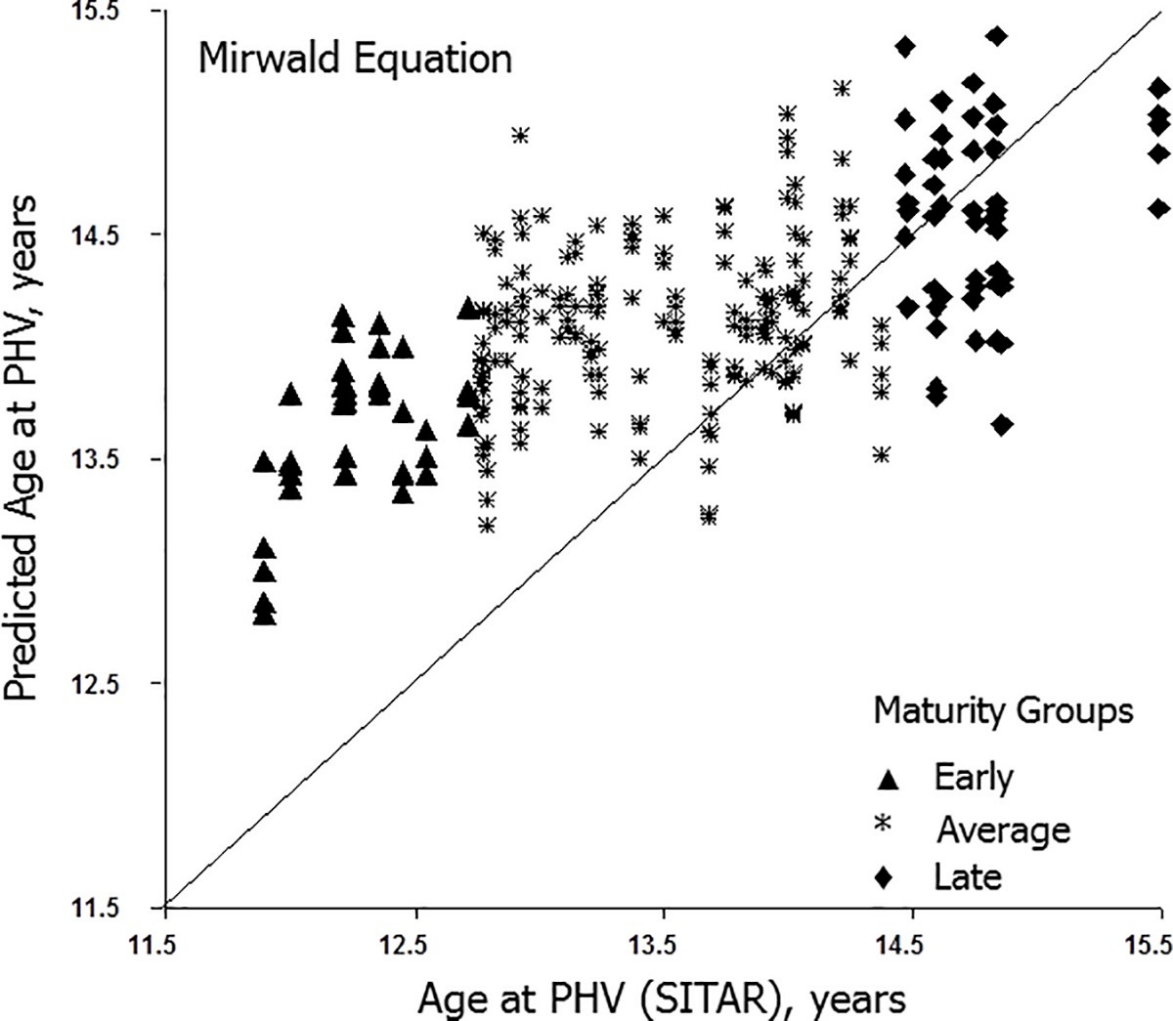
Predicted ages at PHV with the Mirwald equation plotted relative of observed age at PHV at each observation for individual soccer players classified as early, average and late maturing. The diagonal corresponds to the line of identity (x = y).

**Fig 2:**
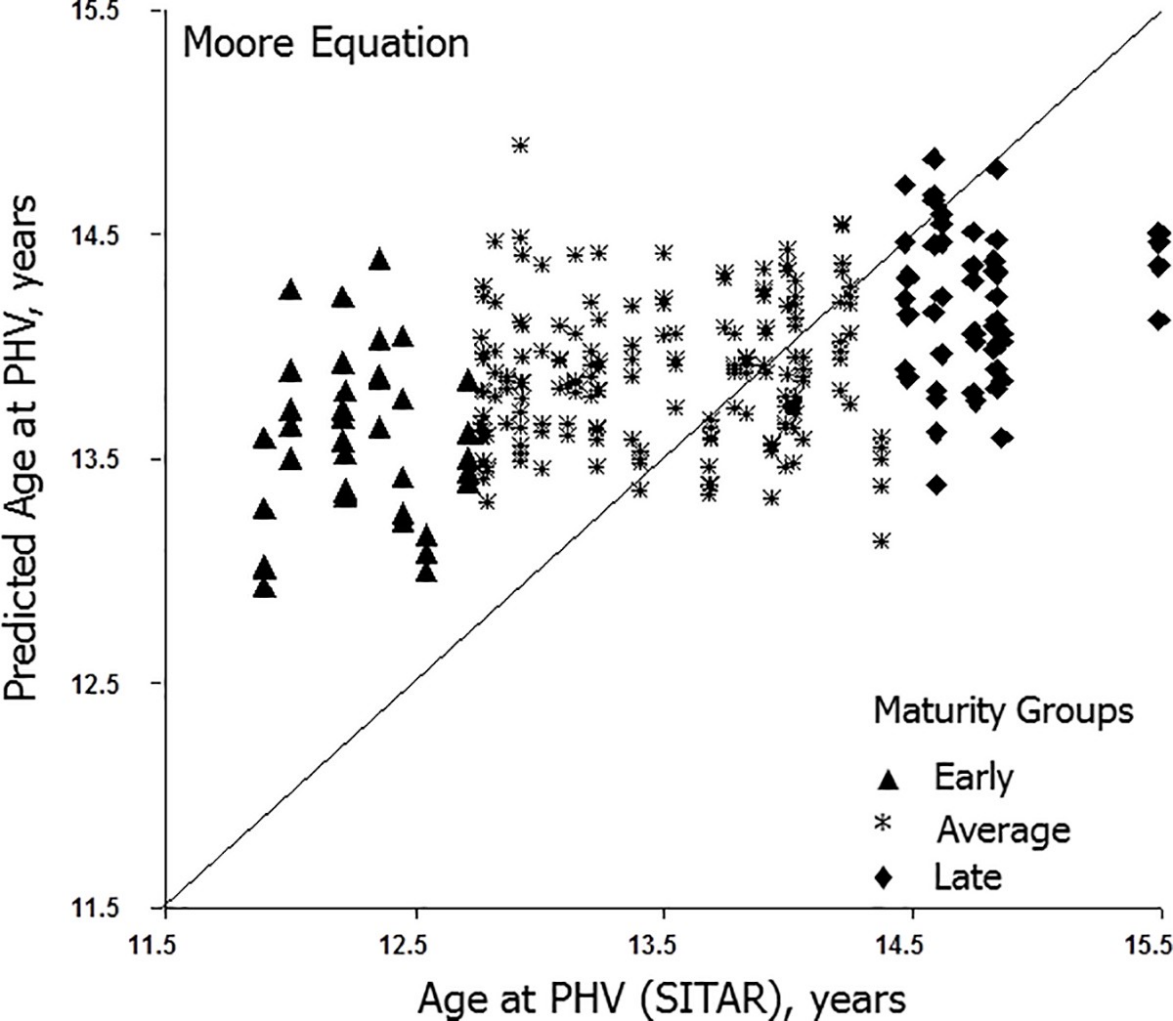
Predicted ages at PHV with the Moore equation plotted relative of observed age at PHV at each observation for individual soccer players classified as early, average and late maturing. The diagonal corresponds to the line of identity (x = y).

Predicted ages at PHV (y-axis) for individual players with the Mirwald and Moore equations are illustrated relative to their respective observed ages at PHV (x-axis) in Figs 1 and 2, respectively. Intra-individual variation in predicted ages at PHV is considerable and ranges of predicted ages are reduced with the Moore equation. Relatively few predicted ages approximate observed ages at PHV in early and late maturing players.

Across the five observations (Table 4), no predicted ages at PHV with the Mirwald equation are within ±0.5 year of observed age at PHV (SITAR) among the eight early maturing players (0 of 39). Corresponding estimates for predicted ages at PHV with the Mirwald equation within ±0.5 year of observed age at PHV across the five observations are 35 of 53 (66%) among late and 76 of 177 (43%) among average maturing players. For the total sample, 111 of 269 (41%) of predicted ages at PHV with the Mirwald equation are within ±0.5 year of observed age at PHV.

**Table 4:** Number of participants by maturity status^†^ according to predicted ages at PHV with the Mirwald equation and, separately, with the Moore equation who were within ±0.50 year of observed age at PHV (SITAR model) at each observation in youth soccer players.

Equation	Observations	Maturity Groups						Total	
		Advanced		Average		Delayed			
		N	n	N	n	N	n	N	n
Mirwald	1	8	0	38	17	12	6	58	23
	2	8	0	38	15	12	8	58	23
	3	8	0	38	18	12	10	58	28
	4	8	0	37	15	10	7	55	22
	5	7	0	26	11	7	4	40	15
	Total	39	0	177	76	53	35	269	111
		0%		43%		66%		41%	
Moore	1	8	0	38	18	12	1	58	19
	2	8	1	38	21	12	5	58	27
	3	8	0	38	19	12	7	58	26
	4	8	0	37	18	10	7	55	25
	5	7	0	26	12	7	3	40	15
	Total (%)	39	1	177	88	53	23	269	112
		3%		50%		43%		42%	

^†^Players were classified as advanced, average or delayed on the basis of their observed age at PHV (SITAR model) relative to age at PHV for the total sample—see text for details.

For the Moore equation, only 1 of 39 predicted ages at PHV (3%) is within ±0.5 year of observed age at PHV among early maturing players. On the other hand, 88 of 177 predicted ages at PHV (50%) among average and 23 of 53 predicted ages at PHV (43%) among late maturing players are within ±0.5 year of observed age at PHV. For the total sample, 112 of 269 (42%) of predicted ages at PHV with the Moore equation are within ±0.5 year of observed age at PHV.

## Discussion

The 58 players comprising the present study was larger than samples in five other longitudinal studies of European youth soccer players, 8 to 33 players [25]. Results of the application of the maturity offset prediction equations in the longitudinal series of Portuguese youth soccer players were consistent with recent studies of English [13] and Dutch [14] soccer players. The study of English players was limited to the Mirwald et al. [1] equation, while that of the Dutch players considered the original and modified [2] equations in addition to an equation which predicted a maturity ratio [50]. Although the three studies varied in design, scope and focus, the results were consistent in highlighting major limitations of predicted maturity offset and predicted age at PHV in longitudinal samples of soccer players.

Applications of the original and modified equations in the longitudinal series of Portuguese youth soccer players were also consistent with validation studies of the maturity offset prediction equations in three longitudinal series of youth spanning late childhood through adolescence, one in the U.S. [9] and two in Poland [10,11]. The three studies and the present study of soccer players used similar analytical methods and noted several major limitations of the prediction equations.

First, predicted maturity offset increased, on average, with CA at prediction throughout the age range considered in each study. In the study of soccer players, mean predicted ages at PHV based on the Moore equation increased, on average, with CA, while those based on the Mirwald equation increased from observation one to two, changed negligibly through observation four and then increased to observation five. The age-related trend probably reflects the predictors comprising each equation which increase, on average, with CA. The preceding is apparent in the correlations between predicted maturity offset and predicted age at PHV with CA, height, sitting height, estimated leg length and weight at each observation for the Mirwald equation and with CA and height with the Moore equation (Table 5). For predicted maturity offset, correlations within each CA group range from moderately high to high; correlations are highest for sitting height and tend to be lowest for estimated leg length. For predicted age at PHV, correlations are relatively low and positive for CA, but are negative and moderate to high for the anthropometric variables. Within an age group, taller and heavier players tended to have an earlier predicted age at PHV.

**Table 5:** Correlations at each observation between predicted maturity offset and predicted APHV (CA–predicted maturity offset) with chronological age (CA), height (Ht), sitting height (SitHt), estimated leg length (LegLt) and body weight (Wt) for the Mirwald equation and with CA and Ht for the Moore equation.

Observations	n	Predicted Maturity Offset							Predicted APHV						
		Mirwald					Moore		Mirwald					Moore	
		CA	Ht	SitHt	LegLt	Wt	CA	Ht	CA	Ht	SitHt	LegLt	Wt	CA	Ht
1	58	0.75	0.85	0.93	0.66	0.81	0.85	0.88	0.30	-0.53	-0.69	-0.34	-0.67	0.31	-0.67
2	58	0.68	0.88	0.94	0.72	0.84	0.80	0.89	0.17‡	-0.70	-0.82	-0.52	-0.80	0.16‡	-0.81
3	58	0.69	0.87	0.96	0.67	0.80	0.78	0.78	0.02‡	-0.82	-0.87	-0.66	-0.87	0.14‡	-0.85
4	55	0.64	0.82	0.93	0.54	0.80	0.77	0.77	0.15‡	-0.74	-0.87	-0.46	-0.80	0.22‡	-0.85
5	40	0.68	0.67	0.85	0.37	0.65	0.81	0.81	0.45	-0.51	-0.77	-0.20‡	-0.65	0.43	-0.77

‡Not significant; all other correlations are significant.

Second, variation in predicted maturity offset and ages at PHV within CA groups was consistently reduced compared to variation in observed ages at offset and at PHV. Variation was reduced more so with the Moore compared to the Mirwald equation.

Third, predictions varied with maturity status defined by observed age at PHV. Predicted maturity offset and ages at PHV were consistently later than observed maturity offset and age at PHV among early maturing boys, and earlier than observed in late maturing boys. By inference, maturity status defined by observed age at PHV influenced predicted ages at PHV in both early and late maturing boys. It should be noted, however, that Moore et al. [2, p. 1761] cautioned that "*Our sample was not large enough to rigorously assess variation in prediction error due to early- and late-maturing children*". This caution, however, is overlooked in applications of the equations. In contrast to early and late maturing youth, predicted ages at PHV appeared to be reasonably accurate for average maturing boys within approximately ±1 year of observed PHV. Unfortunately, the maturity status and/or timing of individuals is not ordinarily known in studies applying the prediction protocols.

Fourth, intra-individual variation in predicted ages at PHV with each equation was considerable in the present sample of soccer players and in each of the longitudinal studies. Ranges of predicted ages at PHV were reduced with the Moore compared to the Mirwald equation.

**Fig 3:**
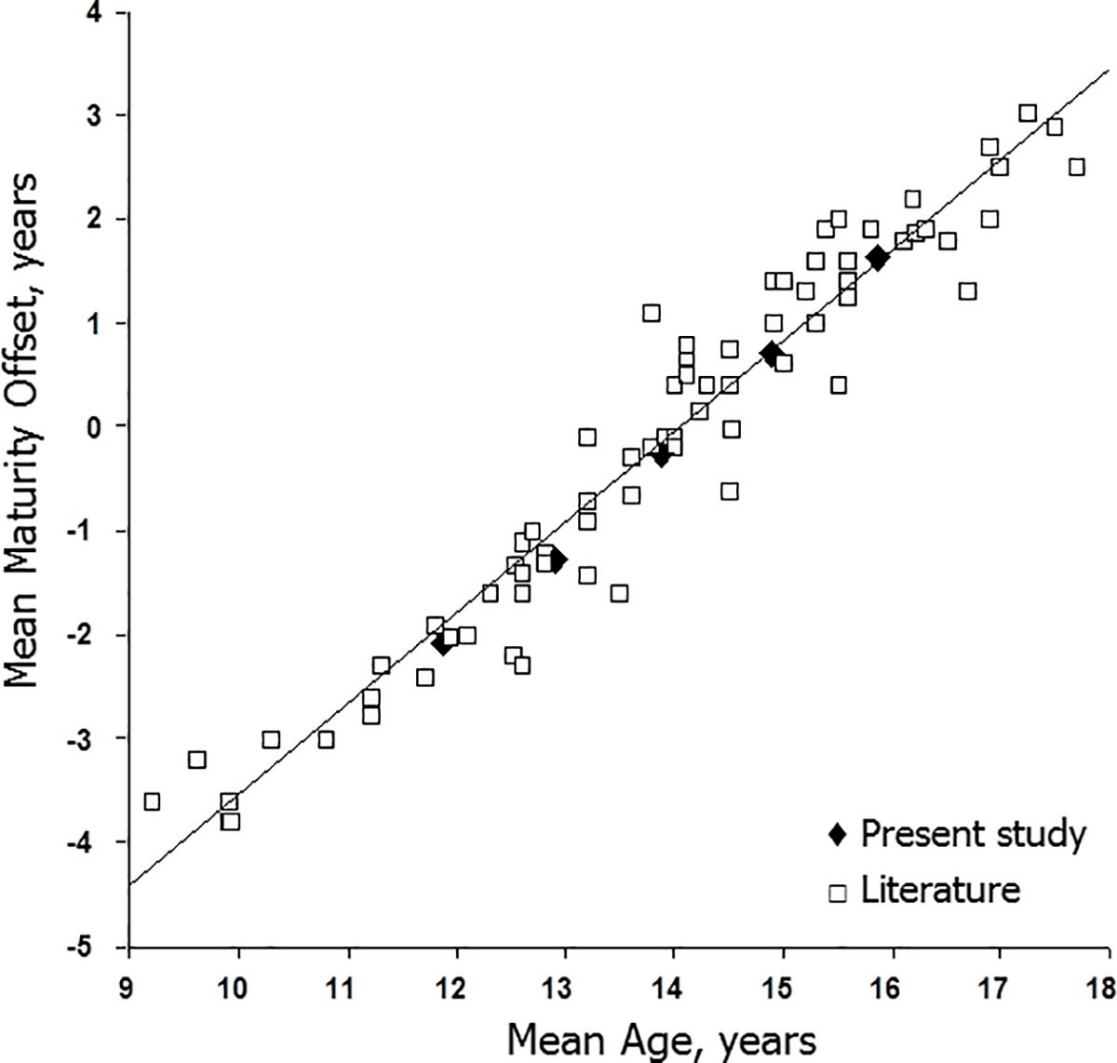
Means for predicted maturity offset (MO) plotted relative to means for chronological age for each year of observation in the current sample of players (filled diamonds) and in samples of male soccer players extracted from the literature (open squares; references: 13, 38, 43, 46, 51–69).

**Fig 4:**
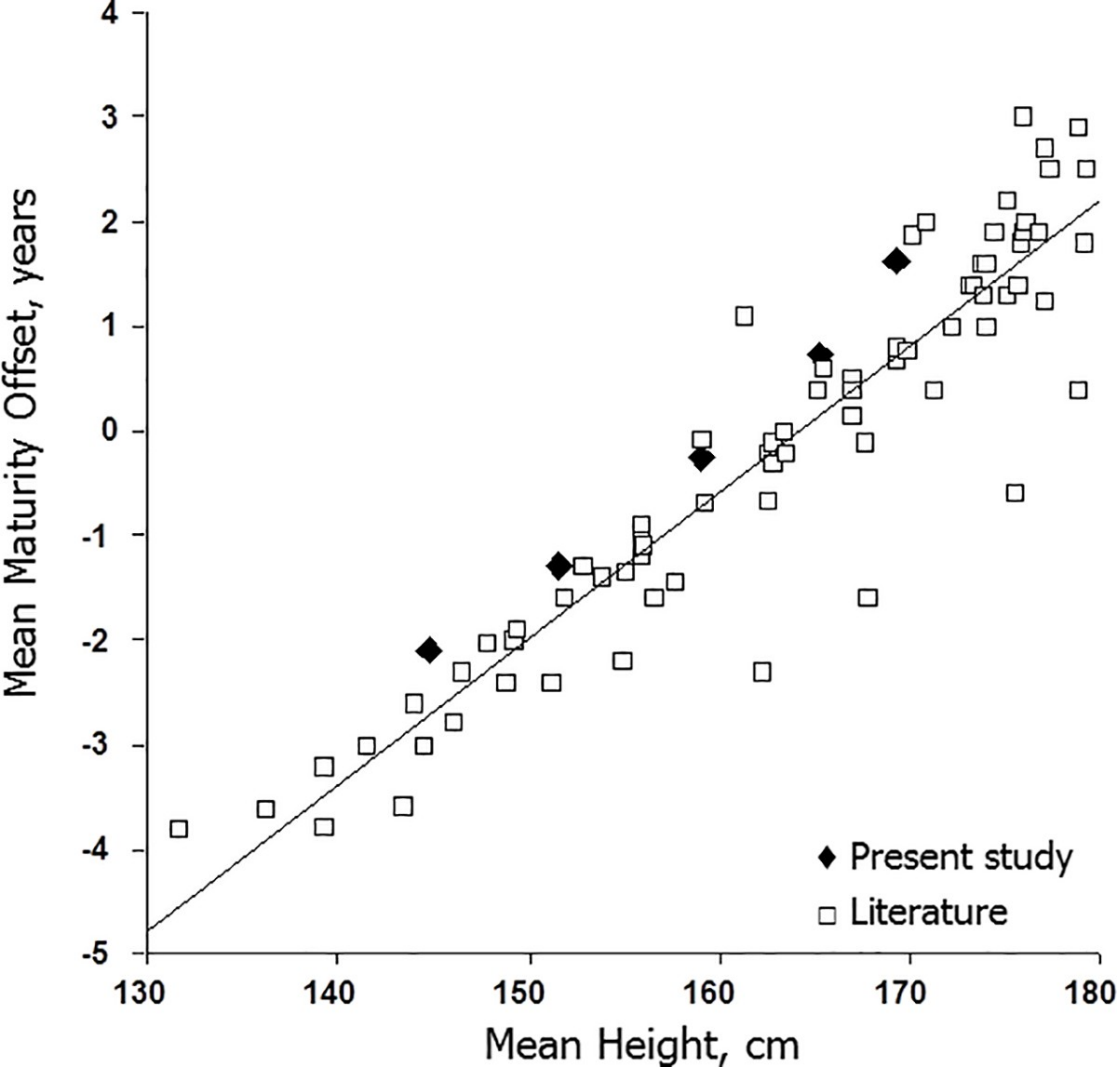
Means for predicted maturity offset (MO) plotted relative to means for height for each year of observation in the current sample of players (filled diamonds) and in samples of male soccer players extracted from the literature (open squares; references: 13, 38, 43, 46, 51–69).

The dependency of predicted maturity offset upon CA and body size at prediction merits attention. Means for predicted maturity offset are plotted relative to means for CA and height in the present study and in samples of male soccer players extracted from the literature [13,38,43,46,51–69] are illustrated in Figs 3 and 4. The means plotted in the figures were limited to studies using the Mirwald equation, as it was more widely used in studies of soccer players. Predicted maturity offset increased linearly with CA and with height at prediction. The plotted means were largely based on one or two year CA groups, although several were based on players spanning age ranges of three or more years.

Studies reporting maturity offset by relative age categories within an age group and studies classifying players across variable age ranges as pre-, at/circa- or post-PHV based on predicted offset were not included in the graphs. In the former, players born early in the year were, on average, older and taller than those born later in the year, while in the latter, CA and height systematically increased from pre-, to at/circa- to post-PHV groups (see above). Given the trends, studies applying predicted maturity offset as a maturity indicator beg the following question: Is predicted maturity offset an indicator of time before age at PHV or is it an indicator of CA and size at prediction? By inference, the validity of predicted maturity offset as an indicator of the time before or after PHV can be questioned.

The preceding has implications for studies using predicted maturity offset as an indicator of maturity status among youth athletes in soccer and other sports. Predicted maturity offset is used most often to classify youth as pre-, at-/circa- and post- PHV, although mean CAs, heights and weights show, on average, a clear gradient across the respective maturity groups. Many studies simply compare the three groups with analysis of variance without controlling for the variation in CA and body size among groups [31,35,36,40,70]. It is also unclear as to how CA-related variation in predicted offset or ages at PHV was addressed in studies applying the prediction equations in short-term longitudinal studies [71–73].

Although studies of youth athletes do not ordinarily indicate the ethnic composition of samples, the issue of ethnic variation is relevant as the maturity offset prediction equations were developed and validated on samples of European ancestry. The original Mirwald equation [1] requires sitting height and estimated leg length, while one of the Moore equations [2] for boys requires age and sitting height. Of potential relevance, population variability in the proportions of sitting height and estimated leg length to standing height is reasonably well established [74,75]. American youth of European (White), African (Black) and Hispanic ancestry, for example, vary in the proportions of sitting height and estimated leg length [76,77]. The proportions of the Portuguese youth soccer players in the present study, as reflected in the sitting height-height ratio, were, on average, generally similar to those for American White and Hispanic youth, but different from American Black youth who have proportionally longer lower extremities. This trend was also noted in a recent study of soccer players in which players of non-European ancestry were taller with a lower sitting height/height ratio, i.e., proportionally longer legs, than players of European ancestry [13].

Results of the current study also have practical implications for those working with youth athletes. The interval of PHV is central to the Long Term Athlete Development (LTAD) model for youth athletes [78,79], which calls for identifying youth of contrasting maturity status, i.e., early, average or late maturing. The LTAD, however, does not specify the method for doing so other than suggesting the monitoring of estimated growth velocities; the latter, however, have limitations over the short term. Estimated increments over short intervals (3–4 months), however, must be interpreted with care; they must be adjusted for the interval between measurements and evaluated relative to factors which influence short term estimates of growth rate–specifically measurement errors (both inter- and intra-observer), diurnal variation and perhaps seasonal variation [80]. Nevertheless, application of predicted maturity offset in this context has the potential for misclassification and thus implications for player development. Those using predicted maturity offset *per se* or variations of the method to identify when players enter and exit the interval of the adolescent growth spurt should employ these methods with caution. If predicted offset is used to inform training design and prescription, it is essential that variation in chronological age at prediction and error associated with the prediction equations be considered. Perhaps additional or alternative methods might be used as a complement, for example, percentage of predicted adult stature attained at the time of observation. As noted above, the utility of estimated velocities of growth in height based on short term height increments has limitations.

The inability of the maturity offset prediction methods to effectively differentiate between early and late maturing youth implies that they should not be used to group players by maturity status as in bio-banding [81], or to adjust fitness and performance scores to accommodate individual differences in maturation [70,82]. As age at PHV is over-estimated in early and under-estimated in late maturing youth, the majority of these players will likely be categorised as being on time and some will be grouped in equivalent bands. Similarly, maturity associated adjustments to performance or fitness scores in early and late maturing boys will, by virtue of these biases, be attenuated and regress towards a common mean.

## Conclusions

Results of the study of applying maturity offset prediction equations to the sample of Portuguese soccer players were consistent with similar studies of soccer players and of the general population. Predicted maturity offset increased, on average, with CA at prediction throughout the age range considered. Variation in predicted maturity offset and ages at PHV within CA groups was consistently reduced compared to variation in observed ages at offset and at PHV. Predictions also varied with maturity status defined by observed age at PHV; predicted maturity offset and ages at PHV were consistently later than observed maturity offset and age at PHV among early maturing boys, and earlier than observed in late maturing boys. And, intra-individual variation in predicted ages at PHV with each equation was considerable and ranges of predicted ages at PHV were reduced more with the Moore compared to the Mirwald equations.

## Supporting information

S1 Table Mixed effects linear models analysis of differences between predicted ages at PHV with the Mirwald (S1A Table) and Moore equations (S1B Table) and observed age at PHV based on the SITAR model, and likelihood ratio tests for differences between two consecutive models (S1C Table).
(DOCX)

## References

[1] Mirwald RL, Baxter-Jones ADG, Bailey DA, Beunen GP. An assessment of maturity from anthropometric measurements. Med Sci Exerc Sports. 2002; 34:689–994. doi: 10.1097/00005768-200204000-00020 11932580

[2] Moore SA, McKay HA, Macdonald H, Nettlefold L, Baxter-Jones ADG, Cameron N, et al. Enhancing a somatic maturity prediction model. Med Sci Sports Exerc. 2015; 47:1755–1764. doi: 10.1249/MSS.0000000000000588 25423445

[3] Malina RM. Top 10 research questions related to growth and maturation of relevance to physical activity, performance, and fitness. Res Q Exerc Sport. 2014; 85:157–173. doi: 10.1080/02701367.2014.897592 25098012

[4] Moran J, Sandercock GR, Ramirez-Campillo R, Meylan C, Collison J, Parry DA. A meta-analysis of maturation-related variation in adolescent boy athletes’ adaptations to short-term resistance training. J Sports Sci. 2017; 35:1041–1051. doi: 10.1080/02640414.2016.1209306 27454545

[5] Moran J, Sandercock G, Rumpf MC, Parry DA. Variation in responses to sprint training in male youth athletes: A meta-analysis. Int J Sports Med. 2017; 38:1–11. doi: 10.1055/s-0042-111439 27793062

[6] Towlson C, Salter J, Ade JD, Enright K, Harper LD, Page RM, et al. Maturity-associated considerations for training load, injury risk, and physical performance in youth soccer: One size does not fit all. J Sport Hlth Sci. 2020; S2095-2546(20)30119-8. doi: 10.1016/j.jshs.2020.09.003 32961300 PMC8343060

[7] Malina RM, Kozieł SM. Validation of maturity offset in a longitudinal sample of Polish boys. J Sports Sci. 2014; 32:424–437. doi: 10.1080/02640414.2013.828850 24016098

[8] Malina RM, Kozieł SM. Validation of maturity offset in a longitudinal sample of Polish girls. J Sports Sci. 2014; 32:1374–1382. doi: 10.1080/02640414.2014.889846 24892233

[9] Malina RM, Choh AC, Czerwinski SA, Chumlea WC. Validation of maturity offset in the Fels Longitudinal Study. Pediat Exerc Sci. 2016; 28:439–455. doi: 10.1123/pes.2015-0090 26757350

[10] Malina RM, Kozieł SM, Králík M, Chrzanowska M, Suder A. Prediction of maturity offset and age at peak height velocity in a longitudinal series of boys and girls. Am J Hum Biol. 2020; Dec 11, e23551. doi: 10.1002/ajhb.23551 33314450

[11] Kozieł SM, Malina RM. Modified maturity offset prediction equations: Validation in independent longitudinal samples of boys and girls. Sports Med. 2018; 48:221–236. doi: 10.1007/s40279-017-0750-y 28608181 PMC5752743

[12] Malina RM, Claessens AL, Van Aken K, Thomis M, Lefevre J, Philippaerts P, et al. Maturity offset in gymnasts: Application of a prediction equation. Med Sci Sports Exerc. 2006; 38:1342–1347. doi: 10.1249/01.mss.0000227321.61964.09 16826033

[13] Parr J, Winwood K, Hodson-Tole E, Deconick FJA, Parry L, Hill JP, et al. Predicting the timing of the peak of the pubertal growth spurt in elite youth soccer players: Evaluation of methods. Ann Hum Biol. 2020; 47:400–408. doi: 10.1080/03014460.2020.1782989 32543933

[14] Teunissen JW, Rommers N, Pion J, Cumming SP, Rössler R, D’Hondt E, et al. Accuracy of maturity prediction equations in individual elite football players. Ann Hum Biol. 2020; 47:409–416. doi: 10.1080/03014460.2020.1783360 32996814

[15] Malina RM, Rogol AD, Cumming SP, Coelho-e-Silva MJ, Figueiredo AJ. Biological maturation of youth athletes: Assessment and implications. Br J Sports Med 2015; 49:852–859. doi: 10.1136/bjsports-2015-094623 26084525

[16] Myburgh GK, Cumming SP, Malina RM. Cross-sectional analysis investigating the concordance of maturity status classifications in elite Caucasian youth tennis players. Sports Med Open. 2019; 5:27. doi: 10.1186/s40798-019-0198-8 31264052 PMC6603099

[17] Malina RM, Coelho-e-Silva MJ, Figueiredo AJ, Carling C, Beunen GP. Interrelationships among invasive and non-invasive indicators of biological maturation in adolescent male soccer players. J Sports Sci. 2012; 30:1705–1717. doi: 10.1080/02640414.2011.639382 22304621

[18] Valente-dos-Santos J, Coelho-e-Silva MJ, Simões F, Figueiredo AJ, Leite N, Elferink-Gemser MT, et al. Modeling developmental changes in functional capacities and soccer-specific skills in male players aged 11–17 years. Pediatr Exerc Sci. 2012; 24: 603–621. doi: 10.1123/pes.24.4.603 23196767

[19] Harris DJ, MacSween A, Atkinson G. Ethical standards in sport and exercise science research: 2020 update. Int J Sports Med. 2019; 40: 813–817. doi: 10.1055/a-1015-3123 31614381

[20] Figueiredo AJ, Gonçalves CE, Coelho e Silva MJ, Malina RM. Youth soccer players, 11–14 years: Maturity, size, function, skill and goal orientation. Ann Hum Biol. 2009; 36:60–73. doi: 10.1080/03014460802570584 19085511

[21] Cao Z, Hui LL, Wong MY. IAPVBS: Individual Age at Peak Velocity Based on SITAR. R package version 0.0.2; 2018. https://rdrr.io/github/Zhiqiangcao/iapvbs/.

[22] Cole T. SITAR: Super Imposition by Translation and Rotation Growth Curve Analysis. R package version 1.1.2; 2020. https://CRAN.R-project.org/package=sitar.

[23] Cole TJ, Donaldson MDC, Ben-Shlomo Y. SITAR–a useful instrument for growth curve analysis. Int J Epidem. 2010; 39:1558–1566. 10.1093/ije/dyq115 PMC2992626 20647267

[24] R Core Team. R: A language and environment for statistical computing. Vienna, R Foundation for Statistical Computing; 2020. https://www.R-project.org.

[25] Malina RM, Coelho-e-Silva MJ, Sousa-e-Silva P, Figueiredo AJ, Cumming SP, Kralik M, et al (under review) Age at peak height velocity in soccer players. 10.1371/journal.pone.0254659 PMC8312932 34310636

[26] Franco-Márquez F, Rodríguez-Rosell D, González-Suárez JM, Pareja-Blanco F, Mora-Custodio R, Yáñez-García, et al. Effects of combined resistance training and plyometrics on physical performance in young soccer players. Int J Sports Med. 2015; 36:906–914. doi: 10.1055/s-0035-1548890 26180903

[27] Hammami R, Granacher U, Marthlouf I, Behm DG, Chaouachi A. Sequencing effects of balance and plyometric training on physical performance in youth soccer athletes. J Strength Cond Res. 2016; 30:3278–3289. doi: 10.1519/JSC.0000000000001425 27144955

[28] John C, Rahlf AL, Hamacher D, Zech A. Influence of biological maturity on static and dynamic postural control among male youth soccer players. Gait Posture. 2019; 68:18–22. doi: 10.1016/j.gaitpost.2018.10.036 30439683

[29] Rodríguez-Rosell D, Franco-Márquez F, Pareja-Blanco F, Mora-Custodio R, Yáñez-Garciá JM, González-Suárez JM, et al. Effects of 6 weeks resistance training combined with plyometric and speed exercises on physical performance of pre-peak height velocity soccer players. Int J Sport Phys Perf. 2016; 11:240–246. doi: 10.1123/ijspp.2015-0176 26218231

[30] Krolo A, Gilic B, Foretic N, Pojskic H, Hammami R, Spasic M, et al. Agility testing in youth football (soccer) players: evaluating reliability, validity and correlates of newly developed testing protocols. Int J Env Res Pub Hlth. 2020; 17:294, 1–15, doi: 10.3390/ijerph17010294 31906269 PMC6981745

[31] Zago M, Moorhead AP, Bertozzi F, Sforza C, Tarabini M, Galli M. Maturity offset affects standing postural control in youth male soccer players. J Biomech. 2020; 99: 109523, 1–5, doi: 10.1016/j.jbiomech.2019.109523 31767282

[32] Pinheiro J, Bates D, DebRoy S, Sarkar D, R Core Team (2020). nlme: Linear and Nonlinear Mixed Effects Models. R package version 3.1–149. https://CRAN.R-project.org/package=nlme.

[33] Cornbleet PJ, Gochman N. Incorrect least-squares regression coefficients in method—comparison analysis. Clin Chem. 1979; 25:432–438. 262186

[34] Lopes Machada DR, Bonfim MR, Costa LT. Pico de velocidade de crescimento como alternative para classificação maturacional associada ao desempenho motor. Rev Brasi Cineantrop Des Hum. 2009; 11:14–21.

[35] Jakovljevic S, Macura M, Radivoj M, Jankovic N, Pajic Z, Erculj F. Biological maturity status and motor performance in fourteen-year-old basketball players. Int J Morphol. 2016; 34:637–643.

[36] Lopez-Plaza D, Alacid F, Muyor JM, Lopez-Minarro PA. Sprint kayaking and canoeing performance based on the relationship between maturity status, anthropometry and physical fitness in young elite paddlers. J Sports Sci. 2017; 35:1083–1090. doi: 10.1080/02640414.2016.1210817 27433884

[37] Read PJ, Oliver JL, de Ste Croix MBA, Myer GD, Lloyd RS. Hopping and landing performance in male youth soccer players: effects of age and maturation. Int J Sports Med. 2017; 38:902–908. doi: 10.1055/s-0043-114009 28931173

[38] Read PJ, Oliver JL, de Ste Croix MBA, Myer GD, Lloyd RS. Landing kinematics in elite male youth soccer players of different chronological ages and stages of maturation. J Ath Train. 2018; 53:372–378. 10.4085/1062-6050-493-16 PMC5967279 29693423

[39] Read PJ, Oliver JL, Myer GD, de Ste Croix MBA, Lloyd RS. The effects of maturation on measures of asymmetry during neuromuscular control tests in elite male youth soccer players. Pediat Exerc Sci. 2018; 30:170–177. doi: 10.1123/pes.2017-0081 28787266 PMC6538932

[40] Peña-González I, Fernández-Fernández J, Cervelló E, Moya-Ramón M. Effect of biological maturation on strength-related adaptations in young soccer players. PloS One, 2019; 14: e0219355. doi: 10.1371/journal.pone.0219355 31276566 PMC6611603

[41] Živković M, Stojiljković N, Antić V, Pavlović L, Stanković N, Jorgić B. The motor abilities of handball players of different biological maturation. Facta Universitatis, series: Physical Education and Sport. 2019; 17:125–133.

[42] Mendez-Villanueva A, Buchheit M, Kuitunen S, Poon TK, Simpson B, Peltola E. Is the relationship between sprinting and maximal aerobic speeds in young soccer players affected by maturation? Pediat Exerc Sci. 2010; 22:497–510. doi: 10.1123/pes.22.4.497 21242600

[43] Buchheit M, Mendez-Villanueva A. Reliability and stability of anthropometric and performance measures in highly-trained young soccer players: effect of age and maturation. J Sports Sci. 2013; 31:1332–1343. doi: 10.1080/02640414.2013.781662 23656211

[44] Cripps AJ, Hopper L, Joyce C. Maturity, physical ability, technical skill and coaches’ perception of semi-elite adolescent Australian footballers. Pediat Exerc Sci. 2016; 28:535–541. doi: 10.1123/pes.2015-0238 27046936

[45] Hammami R, Chaouachi A, Makhlouf I, Granacher U, Behm DG. Associations between balance and muscle strength, power performance in male youth athletes of different maturity status. Pediat Exerc Sci. 2016; 28:521–534. doi: 10.1123/pes.2015-0231 27046937

[46] Morris RO, Jones B, Myers T, Lake J, Emmonds S, Clarke ND, et al. Isometric mid-thigh pull characteristics in elite youth male soccer players: Comparisons by age and maturity offset. J Strength Cond Res. 2020; 34:2947–2955. doi: 10.1519/JSC.0000000000002673 29985220

[47] Asadi A, Ramirez-Campillo R, Arazi H, Saez de Villareal E. The effects of maturation on jumping ability and sprint adaptations to plyometric training in youth soccer players. J Sports Sci. 2018; 36:2405–2411. doi: 10.1080/02640414.2018.1459151 29611771

[48] Brownstein CG, Ball D, Micklewright D, Gibson NV. The effect of maturation on performance during repeated sprints with self-selected versus standardized recovery intervals in youth footballers. Pediat Exerc Sci. 2018; 30:500–505. doi: 10.1123/pes.2017-0240 30033816

[49] Lloyd RS, Oliver JL, Myer GD, de Ste Croix M, Wass J, Read PJ. Comparison of drop jump and tuck jump knee joint kinematics in elite male youth soccer players: Implications for injury risk screening. J Sports Rehab. 2019; 29:760–765. doi: 10.1123/jsr.2019-0077 31629336 PMC9892797

[50] Fransen J, Bush S, Woodcock S, Novak A, Deprez D, Baxter-Jones ADG, et al. Improving the prediction of maturity from anthropometric variables using a maturity ratio. Pediat Exerc Sci. 2018; 30:296–307. 10.1123/pes.2017-0009 28605273

[51] Agostinete RR, Fernandes RA, Narciso PH, Maillane-Vanegas S, Werneck AO, Vlachopolous D. (2020) Categorizing 10 sports according to bone and soft tissue profiles in adolescents. Medicine and Science in Sports 52:2673–2681. doi: 10.1249/MSS.0000000000002420 32735110

[52] Aquino R, Alves IS, Padilha MB, Casanova F, Puggina ER, Maia J. (2017) Multivariate profiles of selected versus non-selected elite youth Brazilian soccer players. Journal of Human Kinetics 60:113–121. doi: 10.1515/hukin-2017-0094 29339991 PMC5765791

[53] Borges PH, Cumming S, Ronque ERV, Cardoso F, Avelar A, Rechenchosky L, et al. (2018) Relationship between tactical performance, somatic maturity and functional capabilities in young soccer players. Journal of Human Kinetics 64:160–169. doi: 10.1515/hukin-2017-0190 30429908 PMC6231338

[54] Campa F, Silva AM, Iannuzzi V, Mascherini G, Benedetti L, Toselli S. (2019) The role of somatic maturation on bioimpedance patterns and body composition in male elite youth soccer players. International Journal of Environmental Research and Public Health 16: 4711, 1–11, doi: 10.3390/ijerph16234711 31779215 PMC6926995

[55] Deprez DN, Fransen J, Boone J, Lenoir M, Philippaerts RM, Vaeyens R. (2015) Characteristics of high-level youth soccer players: variation by playing position. Journal of Sports Sciences 33:243–254. doi: 10.1080/02640414.2014.934707 24998472

[56] Deprez DN, Fransen J, Lenoir M, Philippaerts RM, Vaeyens R. (2015) A retrospective study of anthropometrical, physical fitness, and motor coordination characteristics than influence dropout, contract status, and first-team playing time in high-level soccer players aged eight to eighteen years. Journal of Strength and Conditioning Research 29:1692–1704. doi: 10.1519/JSC.0000000000000806 26010800

[57] Doncaster G, Iga J, Unnithan V. (2018) Assessing differences in cardiorespiratory fitness with respect to maturity status in highly trained youth soccer players. Pediatric Exercise Science 30:216–228. doi: 10.1123/pes.2017-0185 29276855

[58] Gibson NV, Henning G, Twist C. (2019) Movement characteristics, physiological and perceptual responses of elite standard youth football players to different high intensity running drills. Science and Medicine in Football, pp 1–7 doi: 10.1080/24733938.2018.1461235

[59] Gibson NV, McCunn R, MacNay SA, Mullen T, Twist C. (2018) Playing exposure does not affect movement characteristics or physiological responses of elite youth footballers during an intensified period of competition. Science and Medicine in Football, pp 1–6, doi: 10.1080/24733938.2018.1470664

[60] Gill SM, Zabala-Lili, Bidaurrazaga-Leton I, Aduna B, Lekue JA, Santos-Concejero J, et al. (2014) Talent identification and selection process of outfield players and goalkeepers in a professional soccer club. Journal of Sports Sciences 32:1931–1939. doi: 10.1080/02640414.2014.964290 25429718

[61] Gonçalves RR, Severino V, Coelho-e-Silva MJ, Figueiredo AJ. (2011) Age-related variation in anthropometric and maturity characteristics of soccer goalkeepers 11–14 years. Annals of Research in Sport and Physical Activity (University of Coimbra) 1:71–81, doi: 10.14195/2182-7087_1_4

[62] Hernandez Camacho JD, Huelva Leal AB, Martinez-Sanz JM, Lahoz Ruano MD, Vázquez Carrión J. (2018) Peak height velocity and muscle mass in young soccer players. Revista Española de Nutrición Humana y Dietética 22:219–226.

[63] Laas MM, Wright MD, McLaren SJ, Eaves DL, Parkin G, Portas MD. (2020) Motion tracking in young male football players: A preliminary study of within-session movement reliability. Science and Medicine in Football, pp 1–8, doi: 10.1080/24733938.2020.1737329

[64] Lloyd RS, Oliver JL, Radnor JM, Rhodes BC, Faigenbaum AD, Myer GD. (2015) Relationships between functional movement screen scores, maturation and physical performance in young soccer players. Journal of Sports Sciences 33:11–19. doi: 10.1080/02640414.2014.918642 24857046

[65] Lovell TWJ, Bocking CJ, Fransen J, Coutts AJ. (2017) A multidimensional approach to factors influencing playing level and position in a school-based soccer programme. Science and Medicine in Football, pp 1–9, doi: 10.1080/24733938.2017.1420208

[66] Lovell TWJ, Bocking CJ, Fransen J, Kempton T, Coutts AJ. (2017) Factors affecting physical match activity and skill involvement in youth soccer. Science and Medicine in Football, pp 1–8, doi: 10.1080/24733938.2017.1395062

[67] Moreira A, Mortatti A, Aoki M, Arruda A, Freitas C, Carling C. (2013) Role of free testosterone in interpreting physical performance in elite young Brazilian soccer players. Pediatric Exercise Science 25:186–197. doi: 10.1123/pes.25.2.186 23504910

[68] Seabra A, Marques E, Brito J, Krustrup P, Abreu S, Oliveira J, et al. (2012) Muscle strength and soccer practice as major determinants of bone mineral density in adolescents. Joint Bone Spine 79:403–408. doi: 10.1016/j.jbspin.2011.09.003 22071408

[69] Trecoci A, Longo S, Perri E, Iaia FM, Alberti G. (2019) Field-based physical performance of elite and sub-elite middle-adolescent soccer players. Research in Sports Medicine 33:1232–1236. 10.1080/15438627.2018.1504217 30073860

[70] Till K, Jones B. Monitoring anthropometry and fitness using maturity groups within youth rugby league. J Strength Cond Res. 2015; 29:730–736. doi: 10.1519/JSC.0000000000000672 25226333

[71] Mathys SPH, Vaeyens R, Fransen J, Deprez D, Pion J, Vandendriessche J, et al. A longitudinal study of multidimensional performance characteristics related to physical capacities in youth handball. J Sports Sci. 2013; 31:325–334. doi: 10.1080/02640414.2012.733819 23078540

[72] Till K, Cobley S, O’Hara J, Chapman C, Cooke C. A longitudinal evaluation of anthropometric and fitness characteristics in junior rugby league players considering playing position and selection level. J Sci Med Sport. 2013; 16:438–443. doi: 10.1016/j.jsams.2012.09.002 23072898

[73] Zuber C, Zibung M, Conzelmann A. Holistic patterns as an instrument for predicting the performance of promising young soccer players–a 3 year longitudinal study. Front Psychol. 2016; 7:1088. doi: 10.3389/fpsyg.2016.01088 27512378 PMC4961718

[74] Eveleth PB, Tanner JM. Worldwide Variation in Human Growth. Cambridge: Cambridge University Press, 1976.

[75] Eveleth PB, Tanner JM. Worldwide Variation in Human Growth, 2nd edition. Cambridge: Cambridge University Press, 1990.

[76] Malina RM, Brown KH, Zavaleta AN. Relative lower extremity length in Mexican American and in American Black and White youth. Am J Phys Anthropol. 1987; 72:89–94. doi: 10.1002/ajpa.1330720111 3826332

[77] Martorell R, Malina RM, Castillo RO, Mendoza FS, Pawson IG. Body proportions in three ethnic groups: children and youth 2–17 years in NHANES II and HHANES. Hum Biol. 1988; 60:205–222. 3371962

[78] Balyi I, Hamilton A. Long-Term Athlete Development: Trainability in Childhood and Adolescence–Windows of Opportunity, Optimal Trainability. Victoria, BC: National Coaching Institute British Columbia and Advanced Training and Performance Ltd, 2004.

[79] Balyi I, Cardinal C, Higgs C, Norris S, Way R. Canadian Sport for Life: Long-term athlete development resource paper V2. Vancouver, BC: Canadian Sport Centres, 2005; http://www.canadiansportforlife.ca/default.aspx?PageID=1076&LangID=en.

[80] Malina RM, Bouchard C, Bar-Or O. Growth, Maturation, and Physical Activity, 2nd edition. Champaign, IL: Human Kinetics; 2004.

[81] Malina RM, Cumming SP, Rogol AD, Coelho-e-Silva MJ, Figueiredo AJ, Konarski JM, et al. Bio-banding in youth sport: Background, concept, and application. Sports Med. 2019; 49:1671–1685. doi: 10.1007/s40279-019-01166-x 31429034

[82] Abbot S, Hogan C, Castiglioni MT, Yamauchi G, Mitchell LJG, Salter J, et al. Maturity-related developmental inequalities in age-group swimming: The testing of the ‘Mat-CAPS’ for their removal. J Sci Med Sport. 2020; S1440-2440(20)30782-9, doi: 10.1016/j.jsams.2020.10.003 33172611

