# Supplementary material for: Observed and predicted ages at peak height velocity in soccer players
Source: PLoS One. 2021 Jul 26;16(7):e0254659. doi: 10.1371/journal.pone.0254659 (PMC8312932; doi:10.1371/journal.pone.0254659)
Supplement: S1 Table — Mixed effects linear models analysis of differences between predicted ages at PHV with the Mirwald (S1A Table) and Moore equations (S1B Table) and observed age at PHV based on the SITAR model, and likelihood ratio tests for differences between two consecutive models (S1C Table). (DOCX) [file pone.0254659.s001.docx]

**Supplementary Table 1.** Mixed effects linear models analysis of differences between predicted ages at PHV with the Mirwald (Suppl Table 1A) and Moore equations (Suppl Table 1B) and observed age at PHV based on the SITAR model, and likelihood ratio tests for differences between two consecutive models (Suppl Table 1C)

Variables in the analyses:

ID (individual subjects)

D (difference between predicted age at PHV and observed age at PHV)

OBS (consecutive observations: 1-5)

MAT (maturity status: 1= delayed; 2= average, 3= advanced)

The mixed-effects linear models were computed using the Restricted Maximum Likelihood estimator (REML) in the *R*-package *lme4* (Bates et al., 2015). Computation of p-values was based on conditional F-tests with the Kenward-Roger approximation for the degrees of freedom as available in the R-package *pbkrtest* (Halekoh and Hojsgaard 2014). Tables were formatted with the assistance of the *sjPlot* package (Lüdecke, 2021; Nakagawa et al., 2017) and *ggplot2* (Wickham, 2016). Abbreviations in the MLM tables are as follows: CI - 95% confidence intervals, p - significance of the F-test; df - degrees of freedom, Random effects: σ^2^ - random variance estimate; τ_00_ - random intercept variance; τ_11_ - random slope variance; ρ_01_ - random slope-intercept correlation; ICC - intraclass correlation coefficient (proportion of variance explained by a grouping (random) factor - subjects), N - number of groups (subjects); R^2^ - coefficient of determination. Abbreviations for LR test tables: npar - number of parameters; AIC - Akaike Information Criterion; BIC - Bayesian Information Criterion, logLik - log-Likelihood, Chisq - Chi-square statistics, Df - degrees of freedom, Pr(>Chisq) - significance of the Chi-square test.

Bates D, Maechler M, Bolker B, Walker S (2015) Fitting linear mixed-effects models using lme4. J Statistical Software 67(1), 1-48. doi:10.18637/jss.v067.i01

Halekoh U, Hojsgaard S (2014) A Kenward-Roger approximation and parametric bootstrap methods for tests in linear mixed models: The R package pbkrtest. J Statistical Software 59(9), 1-30. URL <http://www.jstatsoft.org/v59/i09/>

Lüdecke D (2021) sjplot: Data visualization for statistics in social science. R package version 2.8.7. URL <https://CRAN.R-project.org/package=sjPlot>

Nakagawa S, Johnson P, Schielzeth H (2017) The coefficient of determination R^2^ and intra-class correlation coefficient from generalized linear mixed-effects models revisited and expanded. J Roy Soc, Interface 14. doi:10.1098/rsif.2017.0213

Wickham H (2016) ggplot2: Elegant graphics for data analysis. Springer-Verlag, New York.

**Supplementary Table 1A**. Results of the linear mixed-effects models analysis of differences between predicted ages at PHV with the Mirwald equation and observed age at PHV

**Model 1.** Unconditional means model.

**Model 2.** Unconditional growth model.

**Model 3.** Model with maturity status (MAT) as fixed factor and interaction with OBS.

**Supplementary Table 1B.** Results of the linear mixed-effects models analysis of differences between predicted ages at PHV with the Moore equation and observed age at PHV

**Model 1.** Unconditional means model.

**Model 2.** Unconditional growth model.

**Model 3a.** Model with maturity status (MAT) as fixed factor and interactions with observations (OBS).

**Model 3b.** Final model with maturity status (MAT) as fixed factor and without interactions.

**Supplementary Table 1C.** Likelihood-ratio tests for differences between two consecutive models

**Mirwald equation**

Each additional factor in the models statistically significantly increased the explained proportion of variance in the data. The best model is Model 3.

**Moore equation**

Each additional factor in the models statistically significantly increased the explained variance in the data. Although Model 3 (with interaction between OBS and MAT) has a slightly lower AIC and BIC than Model 3b (without interaction), the difference is not statistically significant. Therefore, the more parsimonious is Model 3b.
